# Strong Impact of Particle Size Polydispersity on the Thermal Conductivity of Yukawa Crystals

**DOI:** 10.3390/ma17204955

**Published:** 2024-10-10

**Authors:** Konstantin V. Tretiakov, Krzysztof Hyżorek

**Affiliations:** 1 Institute of Molecular Physics, Polish Academy of Sciences, M. Smoluchowskiego 17, 60-179 Poznań, Poland; 2 Faculty of Technology, University of Kalisz, Nowy Świat 4, 62-800 Kalisz, Poland; 3 Game Physics Solutions—Krzysztof Hyżorek, Osiniec 33i, 62-200 Osiniec, Poland; krzysztof.hyzorek@gmail.com

**Keywords:** thermal conductivity, colloid crystal, Yukawa potential, particle size polydispersity, molecular dynamics simulations

## Abstract

Control of thermal transport in colloidal crystals plays an important role in modern technologies. A deeper understanding of the governing heat transport processes in various systems, such as polydisperse colloidal crystals, is required. This study shows how strongly the particle size polydispersity of a model colloidal crystal influences the thermal conductivity. The thermal conductivity of model colloidal crystals has been calculated using molecular dynamics simulations. The model crystals created by particles interacting through Yukawa (screened-Coulomb) interaction are assumed to have a face-centered cubic structure. The influence of the Debye screening length, contact potential, and particle size polydispersity on the thermal conductivity of Yukawa crystals was investigated. It was found that an increase in particle size polydispersity causes a strong—almost fivefold—decrease in the thermal conductivity of Yukawa crystals. In addition, the obtained results showed that the effect of the particle size polydispersity on reducing the thermal conductivity of Yukawa crystals is stronger than changes in values of the Debye screening length or the contact potential.

## 1. Introduction

Interest in colloidal crystals [1] is increasing in light of their possible use in optoelectronics [2], photonics [3], and medicine [4]. To obtain materials with the desired physical properties, research is carried out on colloids composed of spherical and complex molecules, such as “dumbbells” [5], and mixtures of spherical and cylindrical molecules [6]. Electric or magnetic fields are used to control the self-assembly of colloidal crystals [7,8] and to obtain the desired structures by changing the orientation of molecules [8,9]. In condensed-matter physics, the Yukawa potential is widely used to describe the interaction between particles [10]. The screened Coulomb “Yukawa” interaction [11,12] is quite successfully used to describe suspensions of charge-stabilized colloids as long as the standard Derjaguin–Landau–Verwey–Overbeek theory [13] is applicable and the van der Waals interactions are negligible. This repulsive screened-Coulomb potential is a special case of the classical effective pair interaction potential of the single-component colloid model, which describes the interaction between an isolated pair of spherical particles of finite diameter. The particles, which interact through the Yukawa potential with a hard core, form liquid phase and solid phase with body-centered cubic (bcc) or face-centered cubic (fcc) structures depending on the Debye screening length and volume fraction. All those phases were predicted by theory [14,15] and observed both experimentally [16] and in simulations [17,18]. Recently, it has been also shown that Yukawa crystals with a face-centered cubic structure exhibit auxetic properties [19,20].

One of the characteristic features of colloidal systems is the particle size polydispersity [21,22] associated with the synthesis of colloidal particles. That polydispersity deeply affects various thermodynamic properties. An increase in the particle size polydispersity significantly affects the phase diagram of Yukawa systems by shifting the phase transition toward higher packing fractions [23,24]. The influence of polydispersity on the elastic properties of systems [25], in which particles interact through hard [26] and soft (inverse power) [27] interaction potentials, has also been investigated. The influence of size polydispersity on the auxetic properties of Yukawa crystals turned out to be particularly interesting, as it was shown that an increase in size polydispersity leads to the strengthening of the auxetic properties of the system [28]. Further research on the influence of the polydispersity of particle sizes on various physical properties is desirable and may contribute to their possible practical application.

This research is devoted to studying heat transport in a model colloidal system. The thermal conductivity of materials plays an important role in modern technologies. Materials with high thermal conductivity are essential for applications in electronic devices because of their miniaturization, which increases the local power density and leads to temperature growth [29,30]. On the other hand, materials with low thermal conductivity are used in many areas such as clothing, refrigeration, insulation, and thermoelectrics [31]. In bulk materials, high thermal conductivity is mostly observed in crystalline solids with well-defined crystal lattices in which phonons are responsible for heat transport [29,30], whereas disordered and amorphous materials usually have low thermal conductivities. In such materials, the phonon’s mean free path is reduced by increased scattering at the disordered structure and leads to a decrease in thermal transport [32]. In addition, the boundary and interface scattering also affect the thermal conductivity in organic [33] and inorganic [34,35] materials. Regarding colloidal crystals, the geometrical constrictions at the interparticle contact points mainly govern their thermal conductivity, and also the thermal properties of the materials of colloidal particles [36,37]. Most colloidal assembly structures are periodically ordered materials; however, the controlled introduction of the disorder can lead to changes in their physical properties; for instance, in phononic bandgaps in the hypersonic regime [38]. Recently, the influence of structural order on the thermal transport properties of binary mixtures of monodisperse particles with varying size ratios was investigated. It has been shown that disorder in a binary colloidal glass can reduce the thermal conductivity by about 50% compared to the crystalline phase [39]. The question arises as to how the thermal conductivity of a colloidal crystal changes without glass transition while maintaining a regular crystal structure, for example, fcc, but only due to the polydispersity of particle sizes. In other words, how will a local disorder affect the thermal conductivity as opposed to the previously studied effect of global disorder in a binary colloidal glass? A deeper understanding of the impact of local disorder on thermal transport at colloids is important from a practical and theoretical point of view, and Yukawa crystals seem to be an appropriate model system for this purpose. To the best of our knowledge, the effect of varying the size of colloid particles on the thermal conductivity of the polydisperse colloidal crystal has not been investigated so far. This contribution closes this gap and shows how the particle size polydispersity influences the thermal conductivity of model colloidal crystal.

This work’s main aim is to determine the thermal conductivity of the face-centered cubic Yukawa crystals at various Debye screening lengths. In particular, an important aspect of this research is to estimate the effect of particle size polydispersity on the thermal conductivity of Yukawa crystals.

This article is organized as follows. In Section 2, the studied model, the research method, and simulation details are described. Section 3 is devoted to a discussion of the obtained results, whereas Section 4 contains the conclusions.

## 2. Model and Method

### 2.1. Model

We studied a model system which consists of *N* particles that form a face-centered cubic lattice under periodic boundary conditions. An interaction between colloidal particles can be accurately described by models in which particles interact through the hard-core repulsive Yukawa potential (HCRYP) [16,17,18]. This potential combines the hard-core part of the potential responsible for the interaction between the “cores” of colloidal particles associated with their finite size and the Yukawa potential which describes screened-Coulomb repulsion between the charged colloidal particles. In reality, the sterically stabilized, poly-methyl methacrylate colloids [16] can be successfully described by Yukawa’s potential and mimicked by Yukawa crystals.

Original HCRYP [16,17,18] is highly nonharmonic because it contains hard interactions. In molecular dynamics (MDs) simulations, considering the hard interaction requires special treatment [40] that unnecessarily complicates the simulation. Determination of the thermal conductivity coefficient requires long simulation runs to achieve high calculation statistics, and using such a procedure would make the research more difficult. Therefore, part of the potential responsible for the finite size of the particle (hard-core potential) was replaced with an inverse power-law potential with exponent n=96, which was successfully used to describe hard walls [35,41] when determining the thermal conductivity of systems with argon. In the present study, we use an approximation of the HCRYP of the following form

(1) $$\beta u\left(r_{ij}\right)=\beta \varepsilon \frac{\sigma_{i}\sigma_{j}}{\langle \sigma \rangle r_{ij}}e^{-\kappa (r_{ij}-\sigma^{\prime })}+\beta \varepsilon {\left(\frac{\sigma^{\prime }}{r_{ij}}\right)}^{n}$$

where $\sigma_{i}$ and $\sigma_{j}$ are the diameters of interacting particles, $\sigma^{\prime }=\left(\sigma_{i}+\sigma_{j}\right)/2$, and 〈σ〉 is the mean particle diameter in the system, $\beta =1/(k_{B}T)$, *T* is the temperature, $k_{B}$ is the Boltzmann constant, ε is the contact potential, and $k^{-1}$ is the Debye screening length. We consider systems in which particles exhibit dispersion of size that is described by the parameter of polydispersity. The polydispersity is defined as the standard deviation of the particle size distribution divided by the mean [21,23]

(2) $$\delta =\frac{\sqrt{\langle \sigma^{2}\rangle -{\langle \sigma \rangle }^{2}}}{\langle \sigma \rangle }.$$

**Figure 1:**
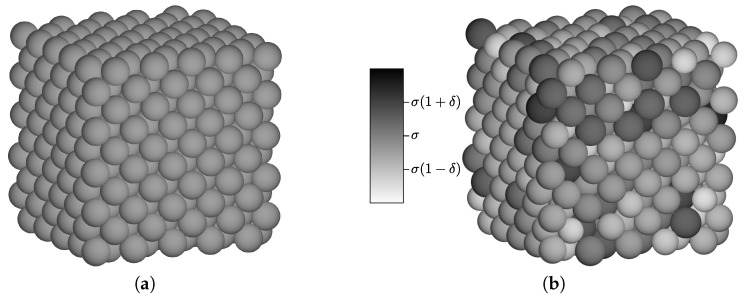
The face-centred cubic Yukawa crystals: (**a**) monodisperse (**b**) polydisperse (with δ=8%).

In real colloidal systems, the distribution of particle diameters can be approximated by Gaussian or log-normal distribution [21,42]. In our work, we took the Gaussian distribution of the diameters of particles as in previous works [21,26,28]. It should be pointed out that for δ=0, we have a system of particles with identical diameters that is further referred to as a monodisperse Yukawa system [17,18,19,20]. The models of face-centered cubic structures of monodisperse and polydisperse Yukawa crystals are shown in Figure 1.

The polydisperse fcc Yukawa crystals with a given polydispersity parameter (δ) were obtained based on structures fulfilling the following conditions

(3) $$\begin{array}{c} \left|\langle \sigma \rangle -1\right|\leq 10^{-5}\;, \end{array}$$

(4) $$\begin{array}{c} \left|\frac{\sqrt{\langle \sigma^{2}\rangle -{\langle \sigma \rangle }^{2}}}{\langle \sigma \rangle }-\delta \right|\leq 10^{-6}.\; \end{array}$$

### 2.2. Method

We determined the thermal conductivity of Yukawa crystals using equilibrium molecular dynamics (EMDs) simulations according to the Green–Kubo formula [43,44,45]. The general form of the Green–Kubo relation, according to the linear response theory [40,46], can be written as follows [40,43,44]

(5) $$K=\int_{0}^{\infty }\langle \dot{A}\left(t\right)\dot{A}\left(0\right)\rangle dt,$$

where *K* is a transport coefficient and $\dot{A}$ is time derivative of a corresponding microscopic variable. The Green–Kubo formula for the thermal conductivity reads as [46]

(6) $$\lambda =\frac{\beta }{VT}\int_{0}^{\infty }\langle j\left(0\right)j\left(t\right)\rangle dt,$$

where *V* is the volume, the angular brackets denote the average over time in the case of a EMDs simulation, and j is the microscopic heat current given as [46,47]

(7) $$j\left(t\right)=\sum_{i}v_{i}\epsilon_{i}+\frac{1}{2}\sum_{i}\sum_{j,i\neq j}r_{ij}\left(F_{ij}\cdot v_{i}\right),$$

where $r_{ij}$ the distance between particles *i* and *j*, $v_{i}$ is the velocity of particle *i*, and $F_{ij}$ is the force exerted on particle *i* due to its neighbor *j* from the pair potential Equation (1). The microscopic site energy (ϵ) is written as

(8) $$\epsilon_{i}=\frac{1}{2}m_{i}{\left|v_{i}\right|}^{2}+\frac{1}{2}\sum_{j,j\neq i}u\left(r_{ij}\right),$$

where *m* is the mass of the particle. In MD simulations, the motion of particles is determined based on the integration of the equations of motion in subsequent simulation steps. To calculate the thermal conductivity, we discretized the right-hand side of Equation (6) in MD time steps as [48]

(9) $$\lambda =\frac{\beta \Delta t}{VT}\sum_{l=1}^{M}\frac{1}{N_{s}-M}\sum_{k=1}^{N_{s}-l}j\left(l+k\right)j\left(k\right),$$

where $N_{s}$ is the number of MD steps after equilibration and *M* is the number of steps over which the time average is calculated. *M* should be considerably smaller than the number of MD steps ($N_{s}$) to ensure good statistical averaging over time.

### 2.3. Simulation Details

MD simulations were performed in the NVT ensemble, where the temperature was controlled by velocity scaling [40]. That choice is based on previous studies in which velocity scaling and a Nosé–Hoover thermostat were used to control the temperature in order to determine the thermal conductivity using the Green–Kubo formula. Those studies showed that the temperature control method does not influence the obtained results that agree with each other [41]. The velocity Verlet algorithm was used to integrate the equations of motion. The integration time step was set to Δt=0.001τ ($\tau =\sqrt{m\sigma^{2}/\varepsilon }$). In calculations, the following independent, dimensionless variables are used: the reduced number density $\rho^{*}=N\sigma^{3}/V$, the reduced pressure $p^{*}=p\beta \sigma^{3}$, and the reduced thermal conductivity $\lambda^{*}=\lambda \sigma \tau /k_{B}$. Simulations were performed for Yukawa systems based on the equation of state from Refs. [18,23] at the following parameters of contact potential βε = 20, 39, 81 and the inverse screening lengths κσ=6.7,10,16.7 at pressure $p^{*}=60$. The present studies have been limited to the parameters of potential for which the fcc Yukawa crystals were observed and close to experimental conditions. For example, the electrostatic potential on the surface of an isolated particle embedded in a low dielectric solvent at room temperature is around 25 mV, which corresponds roughly to βε≈20 [23]. To avoid phase transitions during simulation, the upper limit of the polydispersity parameter was taken δ=0.08 because the terminal polydispersity of studied systems for considered parameters of the Yukawa potential is around 0.095 (9.5%) [23]. The durations of the MD simulation runs were equal to $5\times 10^{6}$ MD steps, after an equilibration of $10^{5}$ MD steps. The number of steps *M* (Equation (9)) over which the time average in the Green–Kubo method was calculated was set to $10^{5}$. All further presented thermal conductivity coefficients are values that were obtained for the thermodynamical limit (N→∞) using a linear approximation based on the results for four sizes of systems with a number of particles equal to *N* = 256, 500, 864, 4000. In addition, each thermal conductivity coefficient at a given thermodynamic condition was determined as the average of ten independent runs.

## 3. Results and Discussion

Charge-stabilized colloidal model crystals with various Debye screening lengths and contact potentials were simulated. We also studied the monodisperse systems in which the colloid particles have the same sizes, and the polydisperse systems in which the colloid particles have different sizes.

### 3.1. Thermal Conductivity of Monodisperse Yukawa Crystals

**Figure 2:**
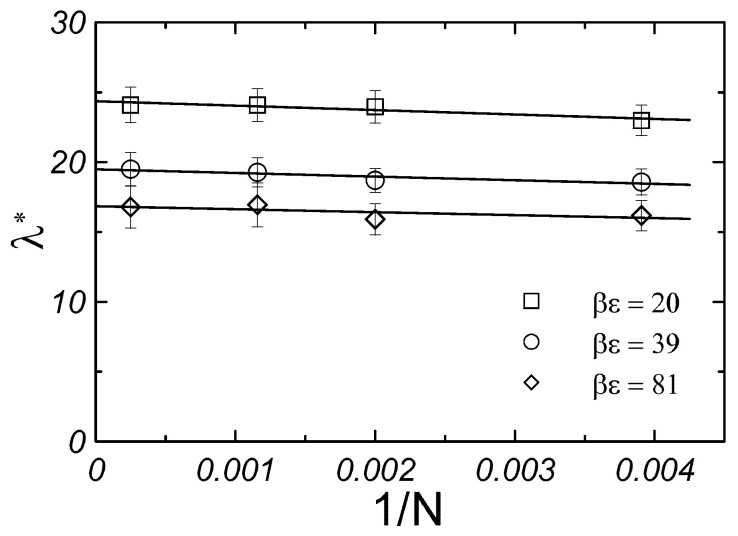
Thermal conductivity of monodisperse Yukawa crystals as a function 1/N at κσ=10 for various values of contact potential.

First, we calculated the thermal conductivity of the monodisperse Yukawa crystals for systems of different sizes and studied the finite-size effect (or size dependence) on the obtained results. We found a weak size dependence similar to that of other systems that were previously studied using the Green–Kubo approach in EMDs simulations [35,41,48,49]. It was observed that for a system of 256 particles, the thermal conductivity of the monodisperse Yukawa crystal differed from λ in the thermodynamic limit by only a few percent (see Figure 2). Regardless of the slight differences in values of thermal conductivities for finite systems and those in the thermodynamic limit, all the thermal conductivity coefficients presented below are given for values in the thermodynamic limit (N→∞).

**Figure 3:**
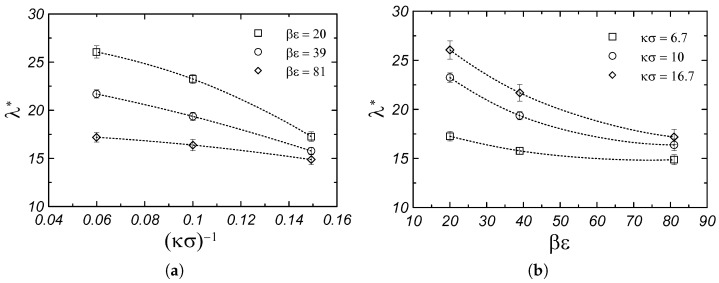
Thermal conductivity of monodisperse fcc Yukawa crystals: (**a**) as a function of Debye screening length and (**b**) as a function of contact potential. The results of the thermodynamic limit (N→∞) are presented. Dotted lines are drawn to guide the eyes.

**Figure 4:**
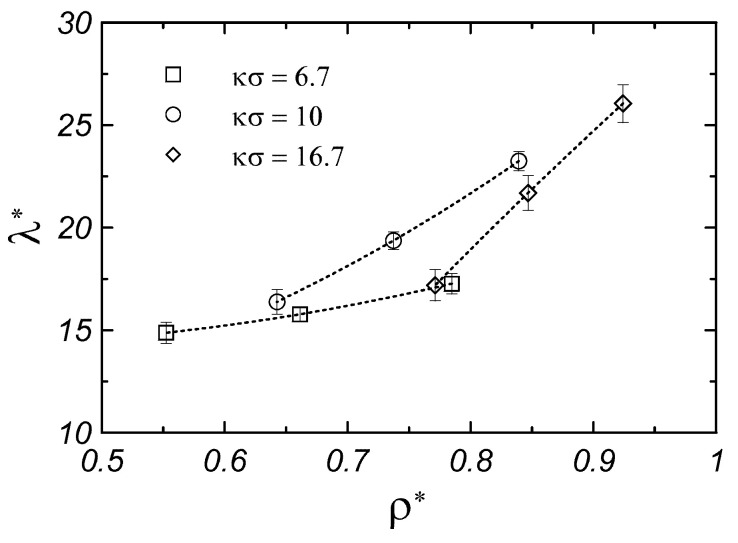
Density dependence of the thermal conductivity of monodisperse fcc Yukawa crystals. Dotted lines are drawn to guide the eyes.

Figure 3 shows the dependence of the thermal conductivity of the monodisperse system on the parameters of the Yukawa potential. From Figure 3a, it can be seen that increasing the screening length, ${\left(\kappa \sigma \right)}^{-1}$, causes a decrease in the thermal conductivity of the system in a wide range of βε. A similar dependence is observed in Figure 3b, where an increase in the value of parameter βϵ causes a decrease in the thermal conductivity of the system. The observed dependences are partly related to the change in the density of the systems (for parameters of Yukawa potential βε and κσ) presented in Figure 4. In the case of model colloidal crystals, increasing the system’s density increases the thermal conductivity. Here, one can add that the parametrization of the Yukawa potential allows us to obtain similar thermal conductivity values for different contact potential values and screening lengths; for example, for κσ=6.7, βϵ=20 and κσ = 16.7, βϵ=81 (see Figure 3). Following the equation of state [18,19] the monodisperse Yukawa systems for those two sets of parameters have similar densities, as shown in Figure 4.

Knowledge of the dependences of the thermal conductivity of the system on the parameters of the Yukawa potential is a reference point for studies regarding the effect of the particle size polydispersity on the thermal conductivity of polydisperse Yukawa crystals.

### 3.2. Thermal Conductivity of Polydisperse Yukawa Crystals

In real colloids, the particle sizes are slightly different. In our studies, this diversity is described by the polydispersity parameter δ (Equation (2)). Based on the phase diagrams determined by Dijkstra and co-authors [23,24] we considered Yukawa crystals with a polydispersity parameter value ranging from 0 to 8%, for which a face-centered cubic structure occurs.

**Figure 5:**
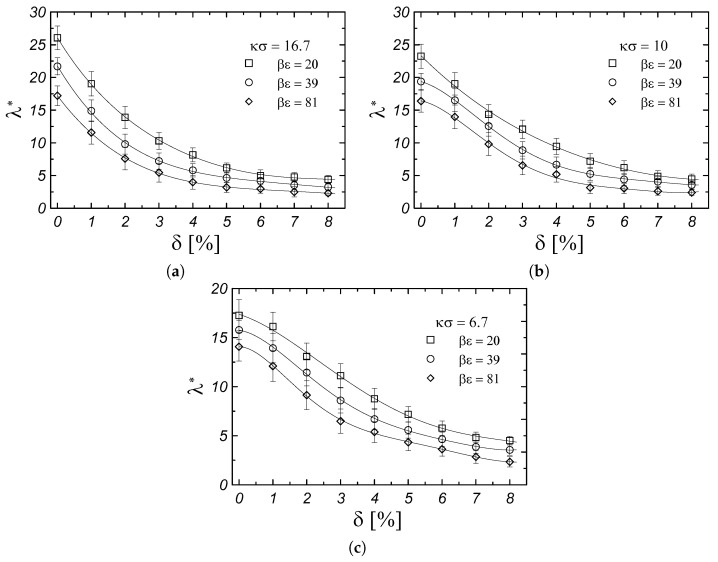
Thermal conductivity of fcc Yukawa crystals as a function of polydispersity for different values of contact potential and screening lengths ${\left(\kappa \sigma \right)}^{-1}$: (**a**) κσ=16.7, (**b**) κσ=10, (**c**) κσ=6.7. Solid lines are drawn to guide the eyes.

Figure 5 shows the effect of particle size polydispersity, δ, on the thermal conductivity coefficient for all studied systems. In all cases, the effect of particle size polydispersity in the system on thermal conductivity is significant and causes a decrease in λ with increasing particle size polydispersity. The strongest effect of particle size polydispersity on the thermal conductivity of crystals is observed for systems with the shortest screening length (Figure 5a). For all systems, increasing the particle size polydispersity to δ=8% causes a four- to fivefold decrease in the thermal conductivity of fcc Yukawa crystals. It is worth noting that the largest considered particle size polydispersity (Figure 5) has a greater effect on the thermal conductivity of the system than the values of the contact potential and screening length in the considered monodisperse systems (Figure 3). Moreover, the change in density with an increase in the polydispersity of particle sizes is only 1% to 3%, depending on the screening length and contact potential. Therefore, the effect of the change in density in the polydisperse system probably makes a negligible contribution to such a strong reduction in thermal conductivity. Recent studies reveal the origin of the low-lattice thermal conductivity in all-inorganic halide perovskites $CsSnBr_{3-x}I_{x}$ [34]. Among other things, one reason for the low thermal conductivity in these materials is the dynamic structural fluctuations that come from a highly dynamic and disordered structure in $CsSnBr_{3-x}I_{x}$. Local disorder due to polydispersity of particle sizes may cause a similar effect, and this could be the subject of further investigation.

## 4. Conclusions

The thermal conductivities of monodisperse and polydisperse Yukawa crystals for a wide range of contact potential values and Debye screening lengths were determined by molecular dynamics simulations using the Green–Kubo method.

Studies involving monodisperse Yukawa crystals showed that an increase in the Debye screening length leads to a reduction in the thermal conductivity. For all the screening lengths considered, the thermal conductivity increases with the density of the crystal. The influence of the crystal density on λ is greater for the shorter screening lengths in the system. Extensive computer simulations have shown that the increase in the size polydispersity of particles in fcc Yukawa crystals leads to a strong decrease in their thermal conductivity. The present results qualitatively confirm recent experimental and theoretical reports on the effect of disorder on the reduction of the thermal conductivity coefficient of colloidal crystals (using a binary mixture as an example) [39]. However, it should be stressed that in the case of the binary mixture, a twofold decrease in the thermal conductivity coefficient associated with the global disorder observed in the glassy state has been found. On the other hand, in the case of polydisperse Yukawa crystals, about a fivefold decrease in the thermal conductivity coefficient was caused by a local disorder, though the regular crystal structure (fcc) was maintained. The origin of such large quantitative differences in the results may suggest the presence of different heat transport mechanisms (or a certain combination of common mechanisms) in polydisperse fcc Yukawa crystals and binary colloidal glass which may be related to local and global disorder, respectively. The above results motivate further research to determine the thermal conductivity coefficients of model amorphous colloidal systems and systems with other crystallographic structures, and special attention should be devoted to studying the mechanisms of heat transport in colloidal crystals. It seems particularly interesting to take into account the variation in particle sizes in these models.

In closing, it is worth mentioning that phononic crystals can control the transport of heat and sound similar to the control of electric currents by semiconductors or light by photonic crystals. It is known that various phononic crystals can be fabricated using self-assembling submicrometer particles into colloidal crystals [50]. Here, it should be stressed that the main advantages of colloidal crystals are their large volume, low cost, and their ability to maintain high-quality translational order [50]. That indicates the importance of the presented studies for a deeper understanding of heat transport processes in colloidal crystals from a theoretical and practical point of view.

## Data Availability

Data is contained within the article.

## Abbreviations

The following abbreviations are used in this manuscript:

MDs	Molecular dynamics
EMD	Equilibrium molecular dynamics
HCRYP	Hard-core repulsive Yukawa potential
fcc	Face-centered cubic
